# Improving the Quality of Two Lives by Treating Obesity

**DOI:** 10.3390/reports8020085

**Published:** 2025-06-03

**Authors:** Norbert Nagy, Patrícia Kleinová, Martin Jozef Péč, Matej Samoš, Ivana Dedinská

**Affiliations:** 1 Department of Internal Medicine I, Jessenius Faculty of Medicine in Martin, Comenius University in Bratislava, 036 01 Martin, Slovakia; norbi.nagy.nn@gmail.com (N.N.); kleinova.pata@gmail.com (P.K.); ivana.dedinska@uniba.sk (I.D.); 2 Department of Transplantation and Nephrology, University Hospital Martin, Kollárova 2, 036 01 Martin, Slovakia

**Keywords:** obesity treatment, living kidney donation, GLP-1 receptor agonist

## Abstract

**Background and Clinical Significance:** Kidney transplantation remains the most effective method of renal replacement therapy. Living donor transplantation offers several advantages—reduced cardiovascular risk, better graft survival, and preemptive intervention. However, donor obesity is a growing concern, as it is usually associated with perioperative and long-term complications, which can affect donor eligibility. Bariatric surgery is a standard recommendation for patients with a BMI over 35 kg/m^2^. There are limited data on the use of pharmacological agents for weight reduction in kidney donors. This case presents a successful conservative treatment with GLP-1 receptor agonist in an obese woman wishing to donate a kidney to her son. **Case Presentation**: We are presenting the case of a 63-year-old woman with grade II obesity who was initially denied being a kidney donor to her son because of her weight. Under these circumstances, she underwent comprehensive lifestyle modification in the cardio-obesitology clinic (caloric restriction, physical activity, and pharmacological treatment with liraglutide). During the 3-month follow-up, she decreased her BMI to 33.4 kg/m^2^, and subsequent examinations confirmed no surgical contraindications to donating a kidney. Despite hematuria, biopsy and genetic testing revealed a benign carrier condition of Alport syndrome, which, without proteinuria or renal impairment, allowed successful kidney donation. **Conclusions**: This case demonstrates that conservative pharmacological treatment for body weight reduction with GLP-1 receptor agonists may be an alternative to bariatric surgery for selected obese kidney donor candidates. The presented case highlights the importance of a multidisciplinary and personalized approach.

## 1. Introduction

The most effective method of replacing kidney function is kidney transplantation. There are two types of donors—living and deceased donors (beating-heart donors and non-beating-heart donors). Kidney transplantation can be performed before starting dialysis treatment—this is known as preemptive transplantation. Only a living donor can perform this kind of transplant in the Slovak Republic. The advantage of this type of transplantation is mainly the elimination of cardiovascular risk resulting from dialysis treatment, with a reduction in mortality of the recipient after transplantation. Other benefits include reduced cold ischemia time and longer graft survival in the post-transplantation period. It is important to remember that if the donor and the recipient are related, a match in the HLA system is a key advantage. The conditions of kidney donation are strict in order to preserve the health of the donor. One of the many criteria is donor obesity, which can cause complications not only on the donor’s side but also on the recipient’s side.

The World Health Organization (WHO) defines obesity as an excessive and abnormal buildup of fat that poses a risk to one’s health. Both adults and children are affected by this pandemic, which is on the rise. The proportion of obese patients over the age of 18 nearly quadrupled (from 7% to 16%) between 1990 and 2022. The rise is considerably more concerning in the pediatric population, which includes those aged 5 to 19 (from 2% to 8%) [1]. According to KDIGO guidelines (2017), patients’ BMI should be computed based on the weight and height of the patient prior to donation and should be classified based on the World Health Organization criteria for the general population. Approval of donor candidates who are obese and have a BMI over 30 should be determined on an individual basis based on their health and demographic profile with respect to the acceptable risk threshold of the transplant program [2].

The aim of this paper is to describe a multidisciplinary approach in the care of a potentially obese living kidney donor in the process of weight reduction, using the case of a 63-year-old female patient.

## 2. Case Report

We present the case of a 63-year-old woman, a potential kidney donor for her son, managed within the interdisciplinary collaboration of two departments of the University Hospital Martin (UNM). The patient’s personal history includes exogenous obesity grade II (BMI 37.8 kg/m^2^), arterial hypertension, status post cholecystectomy, hysterectomy, adnexectomy, and abdominal wall surgery for Schlofer’s tumor. The patient was only taking an angiotensin-converting enzyme inhibitor (perindopril) as the treatment for arterial hypertension. The patient also underwent left knee surgery, followed by reoperation for postoperative complications. The patient was scheduled to be admitted to the UNM Transplant and Nephrology Department in December 2023 for protocol testing prior to potential kidney donation.

Initial laboratory findings included hypertriacylglycerolemia 2.31 mmol/L, borderline fasting glycemia 5.9 mmol/L, vitamin D hypovitaminosis 23.5 μg/L, and urinary sediment showing erythrocyturia 64 μ/L and leukocyturia 42 μ/L; other lab results were not noteworthy. Because of borderline fasting glycaemia, the patient underwent an oral glucose tolerance test, which was suggestive of impaired glucose tolerance. Urine culture revealed the presence of *Escherichia coli* and *Staphylococcus* sp. Coagulase negative at a concentration of 10^2^ in the patient.

A sonographic examination of the abdomen performed while the patient was in the hospital revealed scar-like alterations on the right kidney, worrisome angiomyolipoma of the left kidney, and hepatic steatosis. To rule out the suspicion of angiomyolipoma and as part of the protocol investigations, the patient underwent CT angiography and iliac artery angiography, which did not reveal hemodynamically significant stenosis. The radiologist did not differentiate the suspected angiomyolipoma of the left kidney on the CT scan, as only a small margin of 3 mm was shown on the images obtained. We complemented this with an ultrasonographic examination of the carotid arteries, where we did not find hemodynamically significant stenosis, and a transthoracic echocardiographic examination, where we did not find pathological changes except for type I diastolic dysfunction. We also performed an ophthalmological examination of the patient, which found senile cataract, myopia, astigmatism, and sclerotic changes, evaluated by the specialist as physiological findings due to her age. The gynecological examination was without pathological findings. The patient was contraindicated for surgery by both the transplant nephrologist and the surgeon due to grade II obesity at that time. In view of the above, we conducted an obesitology consultation in the cardio-obesitology outpatient clinic of the Department of Internal Medicine I. After discussion with the patient, we modified the diet, introduced caloric restriction, recommended physical activity, and set the patient on pharmacological treatment with liraglutide once daily subcutaneously. We chose low-calorie, minimally processed foods in the diet, omitting sugary and hyperpalatable foods. The patient did not use any device to track calorie intake or energy expenditure. The interventions to the diet were discussed with the physician during regular check-ups. The patient prepared a list of daily meals with the amounts, and the physician then recommended changes based on the meals the patient ate. For physical activity, we prescribed 150 min of moderate-intensity physical activity per week and daily walks with a goal of 10,000 steps. As the patient lacked previous experience with structured physical activity, we gave general recommendations to promote adherence. During the initial examination, the patient did not report any symptoms or complications related to her previous knee surgery.

We initiated pharmacological treatment from the first examination with liraglutide at a dose of 0.6 mg once daily, with a dose increase of 0.6 mg every week to the highest tolerated dose of 1.8 mg once daily.

After discussion with the patient, we set a follow-up appointment at our outpatient clinic in 3 months, but communication by email was agreed with the patient on a monthly basis to report adverse effects of the treatment and progress with weight reduction. After 3 months of anti-obesity treatment, the patient was scheduled to be readmitted to the Transplantation and Nephrology Department with follow-up at the cardio-obesitology outpatient clinic. After 3 months of the treatment, the patient experienced a weight loss from the initial 116 kg to 102.5 kg, which represents a decrease in BMI to 33.4 kg/m^2^ from the initial 37.8 kg/m^2^ (Table 1). There was normalization of blood pressure, as well as correction of impaired glucose tolerance as evidenced by a controlled oral glucose tolerance test. No chronic medication was changed during this period. Due to the decrease in BMI, the patient was re-evaluated by the transplant nephrologist and surgeon, with the conclusion that weight did not currently present a contraindication to kidney donation. Because of the positive stool examination for occult bleeding in the patient, gastrofibroscopic examination was performed, with the finding of erythematous pangastropathy, while colonoscopic examination was without pathology. A control urine was collected for microscopic examination, and the finding of erythrocyturia persisted despite targeted antibiotic overtreatment. Because erythrocyte typing is unpredictable for the presence of leukocyturia, and also according to KDIGO guidelines 2017, a renal biopsy was performed as part of the differential diagnosis of hematuria. The histopathology result from the kidney biopsy showed the diagnosis of thin basement membrane syndrome. The genetic examination was conducted in the context of the recipients’ anamnesis of Alport syndrome. The genetic testing showed that the donor is an X-linked heterozygote for Alport syndrome. In 95% of European women, this genetic abnormality manifests as microscopic hematuria, without proteinuria, and with physiological renal function; in these cases, the patient can be fully accepted as a kidney donor. After confirming the absence of proteinuria, hypertension, low GFR, and other manifestations of disease, the donor was accepted, and the transplantation was successful.

## 3. Discussion and Literature Review

Opinions vary on the Body Mass Index (BMI) threshold of a living kidney donor. The 2015 European Renal Best Practice (ERBP) recommendations consider a living donor BMI > 35 kg/m^2^ as a contraindication, while the 2020 Kidney Disease Improving Global Outcome (KDIGO) recommendations have more lenient recommendations, with individual consideration of donation in patients with a BMI > 30 kg/m^2^ [3].

Kidney recipients from obese living donors have an increased risk of delayed onset of graft function, prolonged warm ischemia, and impaired graft function. This is due to pathological accumulation of adipose tissue in the renal sinuses and perirenal region, leading to the formation of reactive oxygen species with subsequent activation of proinflammatory and profibrotic processes. Disturbances in serum leptin and adiponectin levels are typical of overweight and obesity. Hyperleptinemia leads to activation of the sympathetic nervous system with a decrease in nitric oxide synthesis. In addition to elevated leptin levels, adipose tissue itself, with compression of the renal parenchyma, leads to activation of the sympathetic and renin-angiotensin-aldosterone system with increased sodium reabsorption, hypertension, and glomerular hypertrophy. Decreased adiponectin levels are associated with the development of insulin resistance and podocyte pedicel smoothing. The named mechanisms lead to structural changes of the graft, which we refer to as obesity-associated glomerulopathy: smoothing of podocyte pedicels, and focal-segmental glomerulosclerosis with glomerular hypertrophy [3].

Obese kidney donors are also subject to complications, the risk of which increases with the degree of obesity. Perioperative complications include: venous thromboembolism, dehiscence and infection of the surgical wound, and the development of lymphocele. In terms of long-term donor survival, meta-analyses have shown a significantly higher risk of post-transplant development of end-stage kidney disease (ESKD) compared to non-obese donors [4]. In view of the above, weight reduction prior to actual kidney donation is desirable [3,4]. The treatment of obesity is complex and includes non-pharmacological, pharmacological, bariatric, and endoscopic treatment. Recently, we have seen a major shift in the pharmacological treatment of obesity; however, this type of treatment is only an adjunctive therapy to non-pharmacological treatment [5]. Pharmacological treatment offers several options: glucagon-like peptide-1 (GLP-1) agonists, orlistat, and naltrexone/bupropion. The indications for their use vary, can be combined, and are individual [6]. Our patient’s goal was to donate a kidney to her son, who suffers from ESKD. At the time of initiation of anti-obesity treatment, the patient’s son was in the CKD KDIGO G5 stage, so we chose the following course of action that would lead to the fastest achievement of the patient’s goal of weight reduction into the overweight or obese stage 1 range. We modified the patient’s diet, reduced carbohydrate and fat intake, and excluded ultra-processed foods and sweetened beverages. We included 150 min of moderate-intensity physical activity, with a daily walking goal of 10,000 steps. Due to her BMI, the patient is also indicated for bariatric surgery; however, due to her preference and lack of time, we chose to treat her with liraglutide initially at a dose of 0.6 mg once daily, with up-titration of the dose weekly to a target of 1.8 mg once daily. We have arranged consultations with the patient once a month, to potentially report adverse effects and monitor the success of the treatment.

The literature on the treatment of the obese kidney donors is lacking. Patients with a BMI above 35 kg/m^2^ are indicated for bariatric surgery. A collective of authors from Brazil describe a decrease of more than 30% in BMI in two patients who underwent bariatric surgery in the period before kidney donation [7]. Other data speak more to the management of obese recipients than donors. Treatment with orlistat and regimen measures in two comparative studies in a group of 201 patients with CKD KDIGO G3-5 resulted in a 6 kg weight reduction in 6 months [8,9]. Another option is the use of a combination agent, naltrexone/bupropion, which led to a reduction of 6.2 kg versus placebo of 1.2 kg in the COR-II trial [10].

GLP-1 analogues are the latest addition to the pharmacological treatment of obesity. In Slovakia, liraglutide is approved for the treatment of obesity. However, semaglutide, dulaglutide, and tirzepatide have also been used in clinical trials for the treatment of obesity. Liraglutide led to significant weight reduction compared to placebo (8.4 kg vs. 2.8 kg), semaglutide had better results (16.1 kg vs. 3.2 kg), while tirzepatide, which is a combined GLP-1 agonist with a glucose-dependent insulinotropic polypeptide agonist, performed best (23.6 kg vs. 2.4 kg) [11,12].

Looking at the results of these data, the question may arise as to why we did not recommend bariatric surgery. In the case of this patient, who underwent preemptive kidney transplantation, we decided to proceed conservatively to ensure rapid weight loss because of the potential operative and postoperative complications, and also because of the patient’s preference. Due to the better results of semaglutide compared to liraglutide, we also considered its use, but due to its unavailability in Slovakia at the initiation of treatment, we finally chose liraglutide.

## 4. Conclusions

Advances in medicine give us great opportunities in the treatment of obesity. There are many drugs in clinical trials with excellent results that could help us in the future in the treatment of potential kidney donors suffering from obesity. Our case report showed that treatment with a GLP-1 receptor analogue can achieve significant weight reduction in a short time, especially if the regimen measures are well set, which allowed us to refer the patient for kidney donation. It can be assumed that the effects of anti-obesity treatment observed in the general population are the same in potential kidney donors, but there are few data to support such a claim, and this is a subject for further investigation.

## Figures and Tables

**Table 1:** Anthropometric parameters of the patient (donor) during the follow-ups.

Our Patient	Body Weight (kg)	Waist Circumference (cm)	Hip Circumference (cm)	BMI (kg/m^2^)
Primary examination	116	110	116	37.8
1. month	112.1	109	114	36.6
2. month	107.5	107	114	35.1
3. month	102.5	104	112	33.4
The difference	−13.5	−6	−4	−4.4

## Data Availability

The original contributions presented in this study are included in the article. Further inquiries can be directed to the corresponding authors.
